# The role of lamivudine versus entecavir in prevention of HCC in patients with hepatitis B virus-related cirrhosis

**DOI:** 10.1186/1471-2334-13-S1-P69

**Published:** 2013-12-16

**Authors:** Cristina Olariu, Adriana Nurciu, Oana Șchiopu, Magdalena Popa, Dan Olteanu

**Affiliations:** 1 National Institute for Infectious Diseases “Prof. Dr. Matei Balş”, Bucharest, Romania; 2 Carol Davila University of Medicine and Pharmacy, Bucharest, Romania; 3 University Emergency Hospital, Bucharest, Romania; 4 Elias University Emergency Hospital, Bucharest, Romania

## Background

In Romania, the incidence of hepatocellular carcinoma (HCC) has increased over the past ten years due to the increasing number of cirrhotic patients (more than 90% of the cases of HCC develop in cirrhosis). Antiviral nucleos(t)ide analogues have been widely used to reduce the development of HCC in chronic hepatitis B patients with fibrosis or cirrhosis.

## Methods

We included 36 patients with hepatitis B virus related cirrhosis. Since February 2008 until now, 45% (16 patients) were treated with lamivudine and 55% (20 patients) with entecavir. The evaluation was performed every 3-4 months by clinical exam, biochemical and serological tests, and abdominal ultrasonography for all patients. Upper endoscopy, computed tomography scanning or magnetic resonance imaging were done in selective cases.

## Results

The median age at the diagnosis of cirrhosis was 57.6±10.7 years and 75% of patients were males. Six cases (16.66%) were diagnosed with HCC – all of them were males. Only one of these patients was treated with entecavir, while the rest received lamivudine. Risk factors involved in carcinogenesis were represented by: age over 60, male gender, cirrhosis evolution for more than 5 years.

## Conclusion

The results of this study suggested that the incidence of HCC is lower in patients with HBV-related cirrhosis who used entecavir (5%) compared to lamivudine (31%).

